# Evolution of Asian Interior Arid-Zone Biota: Evidence from the Diversification of Asian *Zygophyllum* (Zygophyllaceae)

**DOI:** 10.1371/journal.pone.0138697

**Published:** 2015-09-22

**Authors:** Sheng-Dan Wu, Li Lin, Hong-Lei Li, Sheng-Xiang Yu, Lin-Jing Zhang, Wei Wang

**Affiliations:** 1 College of Life Sciences, Shanxi Normal University, Linfen, China; 2 State Key Laboratory of Systematic and Evolutionary Botany, Institute of Botany, Chinese Academy of Sciences, Beijing, China; 3 Key Laboratory of Southern Subtropical Plant Diversity, Fairylake Botanical Garden, Shenzhen & Chinese Academy of Sciences, Shenzhen, China; The National Orchid Conservation Center of China; The Orchid Conservation & Research Center of Shenzhen, CHINA

## Abstract

The Asian interior arid zone is the largest desert landform system in the Northern Hemisphere, and has high biodiversity. Little is currently known about the evolutionary history of its biota. In this study, we used *Zygophyllum*, an important and characteristic component of the Asian interior arid zone, to provide new insights into the evolution of this biota. By greatly enlarged taxon sampling, we present the phylogenetic analysis of Asian *Zygophyllum* based on two plastid and one nuclear markers. Our phylogenetic analyses indicate that Asian *Zygophyllum* and *Sarcozygium* form a clade and *Sarcozygium* is further embedded within the shrub subclade. An integration of phylogenetic, biogeographic, and molecular dating methods indicates that *Zygophyllum* successfully colonized the Asian interior from Africa in the early Oligocene, and Asian *Zygophyllum* became differentiated in the early Miocene and underwent a burst of diversification in the late Miocene associated with the expansion of Asian interior arid lands due to orogenetic and climatic changes. Combining diversification patterns of other important components of the Asian interior arid zone, we propose a multi-stage evolution model for this biota: the late Eocene–early Oligocene origin, the early Miocene expansion, and the middle-late Miocene rapid expansion to the whole Asian interior arid zone. This study also demonstrates that, for *Zygophyllum* and perhaps other arid-adapted organisms, arid biomes are evolutionary cradles of diversity.

## Introduction

Over the surface of the Earth, biodiversity is not evenly distributed, but is clustered into several biomes. Understanding the origin and evolution of biomes is a fundamental issue in biology and ecology [1]. However, biomes harbor many species, each of which has an individual evolutionary history. As reconstructing the evolutionary histories of all species in a biome is not realistic, inferring the timing and tempo of diversification in biome-specific plant groups offers a possible means of investigating the historical construction of the biome that they characterize [2,3]. For example, Palmae and Menispermaceae have been used as indicators to track tropical rainforests through time [4,5]. Arid lands occupy about one-third of the Earth’s land surface [6,7], and harbor abundant arid-adapted organisms [8]. Owing to extreme arid and enormous difference between daytime and nighttime temperatures, arid biomes are very fragile and sensitive to climatic changes. Thus, an estimation of the evolutionary dynamics of arid-land biodiversity is vital and urgent for the conservation of these areas, and can predict how they will respond to future climate changes [9,10].

Based on geographic latitudes, Walter [11] divided arid zones into two types: the tropical and subtropical arid zone (0°–30°), occurring in Africa, Australia, and the New World, and the temperate arid zone (>30°), mainly limited to the Asian interior. There have been many studies focusing on the origin and evolution of arid biomes in Africa [12,13], Australia [14,15], and the New World [16,17]. Additionally, Loera et al. [18] and Töpel et al. [19] explored diversification in Northern American arid lands. These studies indicate that different tropical and subtropical arid biomes most likely originated in response to aridification trends during the Neogene. The temperate Asian arid lands are distributed in the interior of Eurasia and consist of the largest arid zone in the Northern Hemisphere [20]. However, little is known about the evolutionary history of the biota of this arid zone.

Asian arid inlands make up the desert regions in Irano-Turania, central Asia, northwestern China, and Mongolia [21–23]. These arid regions are characterized by extreme winter cold and year-round low precipitation (less than 200 mm annually) [11]. In spite of the unfavorable environment, the arid Asian interior contains high biodiversity. In the preliminary inventory of this arid zone, Hu et al. [8] listed 127 families and 1279 genera of angiosperms. The origin and evolution of the Asian arid interior have fascinated botanists and geologists [24,25]. During the past four decades, tremendous progress has been made in understanding the aridification process of the Asian inland region [24,26]. Based on eolian deposition, Asian interior desertification occurred during the late Oligocene—the early Miocene (22–25 million years ago, Ma) [27–29], and significantly intensified during recent 3–4 Ma since the Pliocene [30,31]. Nevertheless, the emergence of novel environmental conditions in a region may not be synchronous with the colonization of a habitat by a given lineage, i.e., there is sometimes an evolutionary lag time [32]. Importantly, because of the difficulty in obtaining specimens from central Asia, studies of the diversification of organisms inhabiting the Asian interior arid zone are relatively rare. Current studies mainly focus on arid Northwest China and are at the population level ([25] and references therein), which only elucidate the biotic evolution of arid Northwest China since the Quaternary. Thus, our knowledge about the evolution of the Asian interior arid-zone biota remains incomplete so far.

**Fig 1:**
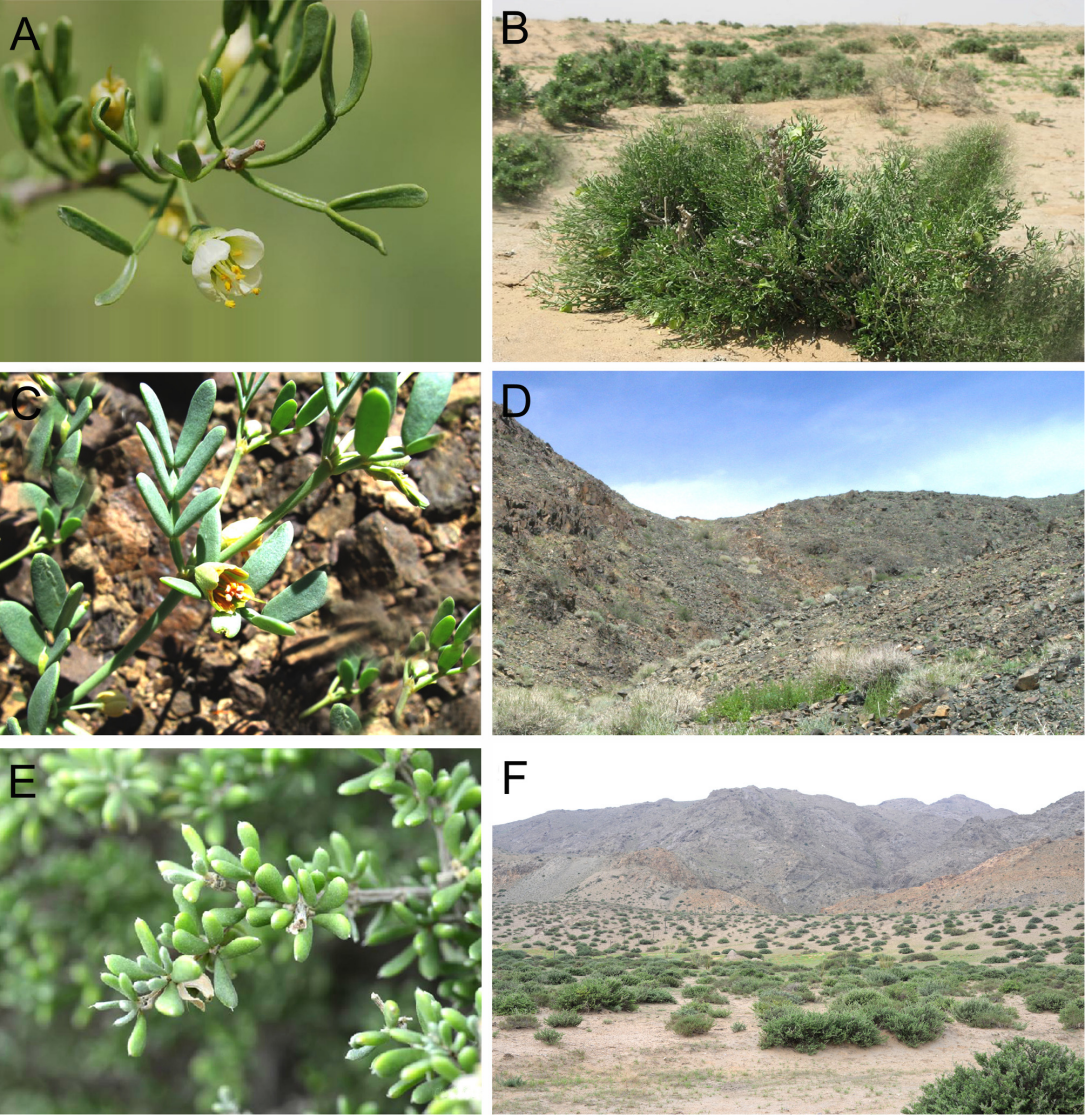
Examples of Asian interior arid-zone vegetation dominated by *Zygophyllum* and *Tetraena*. *Zygophyllum xanthoxylon* (A) and its community (B); *Zygophyllum pterocarpum* (C) and its community (D); *Tetraena mongolica* (E) and its community (F). Photographs by S. Yu.

In this study, we inferred the origin and evolution of the Asian interior arid-zone biota by examining the history of the diversification of Asian *Zygophyllum* (Zygophyllaceae). *Zygophyllum* is distributed in arid regions of Africa, Australia, and Asia [33]. In Asian inland arid ecosystems, *Zygophyllum* plants are among the most important and characteristic components in terms of their contribution to the vegetation and impact on the environment (Fig 1a–1d; [34]). Approximately 51 species of *Zygophyllum* are found in the Asian interior [35]. Based on the literature ([35] and references therein) and our examination of herbarium specimens, almost all of these 51 species are restricted to arid regions, with a few extending to neighboring regions. Additionally, phylogenetic studies have placed the monotypic genus *Tetraena* in *Zygophyllum* [35], *Tetraena* is endemic to Inner Mongolia, China, and is also considered to be a key representative of arid vegetation (Fig 1e and 1f; [36]). Morphological and anatomical features indicate that *Zygophyllum* and *Tetraena* plants can efficiently use water and are well adapted to arid habitats [37,38]. Moreover, *Zygophyllum* plants use the C_4_ photosynthetic pathway [39,40], which is advantageous in conditions of drought, sun burn, and high temperature [41]. On the basis of the older bound of a BEAST estimate using uniformly distributed constraints, Bellstedt et al. [42] suggested that Asian *Zygophyllum* and *Tetraena* could have originated in the Eocene and Miocene, respectively. Thus, Asian *Zygophyllum* and *Tetraena* constitute an ideal model group to study the diversification of the Asian interior arid-zone biota.

Of the 51 Asian *Zygophyllum* species, less than nine species have been sampled in previous studies [35,42–44]. The objectives of this study are to (1) reconstruct a phylogenetic framework for Asian *Zygophyllum* using three molecular markers from nuclear and plastid genomes with a more extensive sampling than in any previous study, (2) to investigate the diversification dynamics of Asian *Zygophyllum* over time by integrating phylogenetic, molecular dating and biogeographic methods, and (3) to explore the evolution of the Asian interior arid-zone biota using Asian *Zygophyllum* as a model group.

## Materials and Methods

### Taxon sampling

In this study, we followed the system of Sheahan [33], in which *Zygophyllum* includes *Sarcozygium*. We sampled 24 accessions, representing 23 Asian *Zygophyllum* species and including the type species of *Zygophyllum* and *Sarcozygium*, *Zygophyllum fabago* L. and *Sarcozygium xanthoxylum* Bunge (= *Zygophyllum xanthoxylum* (Bunge) Engl.). Our taxon sampling scheme covers the whole Asian arid land and contains a morphological diversity of Asian *Zygophyllum* [33,35]. *Zygophyllum*, *Augea*, *Fagonia*, and *Tetraena* make up the Zygophylloideae [33]. However, phylogenetic analyses have indicated that *Zygophyllum* is not monophyletic and includes *Augea* from Africa, *Fagonia* from Africa and the New World, and *Tetraena* from China [35,42–44]. In the Zygophylloideae, six major clades, *Augea*, *Fagonia*, *Melocarpum*, *Tetraena*, *Zygophyllum*, and *Roepera*, are recognized [42]. To explore the evolutionary history of Asian *Zygophyllum*, we also sampled 26 species of the *Tetraena* clade (including *Tetraena mongolica*), 2 species of the *Augea* clade, 32 of the *Fagonia* clade, 2 species of the *Melocarpum* clade, and 41 species of the *Roepera* clade. Following the previous results [35], we selected *Guaiacum angustifolium* and *Larrea tridentata* (Larreoideae), and *Tribulus terrestris* (Tribuloideae) as outgroups. Voucher information and GenBank accession numbers are listed in S1 Table.

### DNA extraction, PCR amplification and sequencing

Three molecular markers were used in this study: plastid *trnL* intron and *trnL* [UAA] 3’ exon*-trnF* [GAA] intergenic spacer and nuclear ribosomal internal transcribed spacer (nrITS). Total DNA was extracted from silica-gel-dried leaf material using DNA Extraction Kit for GMO Detection (TaKaRa Biotechnology, Dalian, China) following the manufacturer’s protocol. The selected DNA regions were amplified using standard polymerase chain reaction (PCR). The ITS, *trnL* intron, and *trnL-F* spacer regions were amplified and sequenced using the primers ITS-F1 (5’-GTC CCA TTC TAT ATG TCA GT-3’) and ITS-R1 (5’-CCC CAC GAT TTC TAA AGT CGA CG-3’), c and d [45], and e and f [45], respectively. PCR amplifications were performed in 25 μl reactions with the following thermocycler program: 2 min at 95°C for denaturation, then 35 cycles of 30 s at 95°C, 30–50 s at 52–56°C for annealing, 1 min 30 s at 72°C for primer extension, and a 10-min incubation at 72°C following the cycles. The standard 25 μl PCR reaction mix consisted of 2 mM MgCl_2_, 200 μM dNTPs, 1 pM primer, 0.025 U/μL Taq polymerase, 1–2 μL DNA, and a reaction buffer provided by TaKaRa GMO Rapid Screening Kit (TaKaRa Biotechnology, Dalian, China). The PCR products were purified using TaKaRa Agarose Gel DNA purification Kit version 2.0 (TaKaRa Biotechnology, Dalian, China). Sequencing reactions were conducted using an ABI Prism BigDye Terminator Cycle Sequencing Kit (Applied Biosystems, ABI, BJ, China). Sequences were analyzed using ABI 3730 × l DNA Analysis Systems following the manufacturer’s protocols.

### Phylogenetic analysis

The sequences of each locus were aligned and manually adjusted in Geneious version 6.0 [46]. We first used maximum likelihood (ML) method to perform nonparametric bootstrap analyses for individual loci. No significant bootstrap support for incongruent nodes was evident (exceeding 70%) among the three loci, and the individual data sets were therefore combined. The ML and Bayesian inference (BI) methods were used to analyze the combined data set. ML analyses were performed in the CIPRES (Cyberinfrastructure for Phylogenetic Research; www.phylo.org) with RAxML-HPC2 on XSEDE [47,48]. RAxML was conducted using a GTR + Γ substitution model for each marker, and the fast bootstrap option, using 1000 replicates. For BI analysis, each DNA region was assigned its own best-fit model, as determined by the Akaike Information Criterion (AIC) using jModelTest version 2.1.4 [49]. All three partitions used GTR + Γ model. BI analysis was performed using MrBayes version 3.1.2 [50]. Four Markov chain Monte Carlo (MCMC) chains (three heated and one cold chain, temperature of 0.2) were run, sampling one tree every 1000 generations for 10,000,000 generations, starting from random trees. We used Tracer version 1.6 [51] to assess the stationarity of the runs. A majority-rule (>50%) consensus tree was constructed after removing the burn-in period samples (initial 25% of the sampled trees). The posterior probability (PP) was used to estimate nodal robustness.

### Molecular dating

Divergence times were estimated in BEAST version 1.8.0 [52], which employs a Bayesian MCMC approach to co-estimate topology, substitution rates and node ages. All dating runs were conducted using the GTR + Γ + I model (with eight rate categories), a Yule tree prior, with rate variation across branches uncorrelated and lognormally distributed. The MCMC chains were run for 50 million generations, with sampling every 1000 generations. Tracer version 1.6 [51] was used to check appropriate burn-in and the adequate effective sample sizes of the posterior distribution (>200). The resulting maximum clade credibility trees were edited in FigTree version 1.3.1 (http://tree.bio.ed.as.uk/software/figtree/).

The Zygophyllaceae fossil record is both sparse and can not be confidently placed in the tree of extant taxa (reviewed by Bellstedt et al. [42]). We therefore used a two-pronged approach to estimate divergence times. Asian *Zygophyllum* and *Tetraena* are distributed in different clades in the Zygophylloideae [35,42,44]. First, we estimated the stem- and crown-group ages for the Zygophylloideae using plastid *rbcL* sequences from 45 Zygophyllaceae and Krameriaceae (the sister group to Zygophyllaceae) species (S2 Table). These selected species represent all six clades of Zygophylloideae and other four subfamilies of Zygophyllaceae. *Viscainoa geniculata* and *Malesherbia paniculata* (Fabidae) and *Geranium cuneatum* subsp. *tridens* (Malvidae) were chosen as outgroups. Following the results of Bell et al. [53], the split between Zygophyllaceae and Krameriaceae was set at 70 Ma (49–88 Ma) and the crown-group age of Fabidae was set at 107 Ma (101–114 Ma). The stem and crown group ages of Zygophylloideae, inferred from the *rbcL* dataset, were used as secondary calibration points (using normal prior distributions) in the Zygophylloideae-centered BEAST analysis. We also used 60.9 Ma (34–90 Ma) to constrain the stem age of Zygophyllaceae, recently produced by Magallón et al. [54]. These two constraints generated highly congruent results.

For the purpose of comparison and confirmation, we further estimated the divergence times using an expanded ITS dataset (with sequences from GenBank added to our own; see S3 Table). The stem age of Zygophyllaceae was set at 70 Ma (49–88 Ma) [53] to infer ages of Zygophylloideae and Asian *Zygophyllum*. Ages inferred from the expanded ITS dataset largely agreed with those inferred by using the above two-pronged approach.

### Biogeographic analysis

To reconstruct the ancestral areas of Asian *Zygophyllum*, we used the Statistical Dispersal-Vicariance analysis (S-DIVA) in the software package RASP [55]. Using the posterior distribution of trees resulting from a BEAST analysis and generating credibility support values for alternative phylogenetic relationships, S-DIVA can minimize the uncertainties associated with phylogenetic inference [56,57]. We randomly sampled 1000 trees from the BEAST output as a “tree file” and used the maximum clade credibility tree as a final representative tree. Based on the distribution of *Zygophyllum* and allies, four geographic regions were coded: A, Africa; B, Asia; C, Australia; and D, New World. Areas were delimited based on Beier et al. [35] and Bellstedt et al. [42].

### Diversification rate analysis

**Fig 2:**
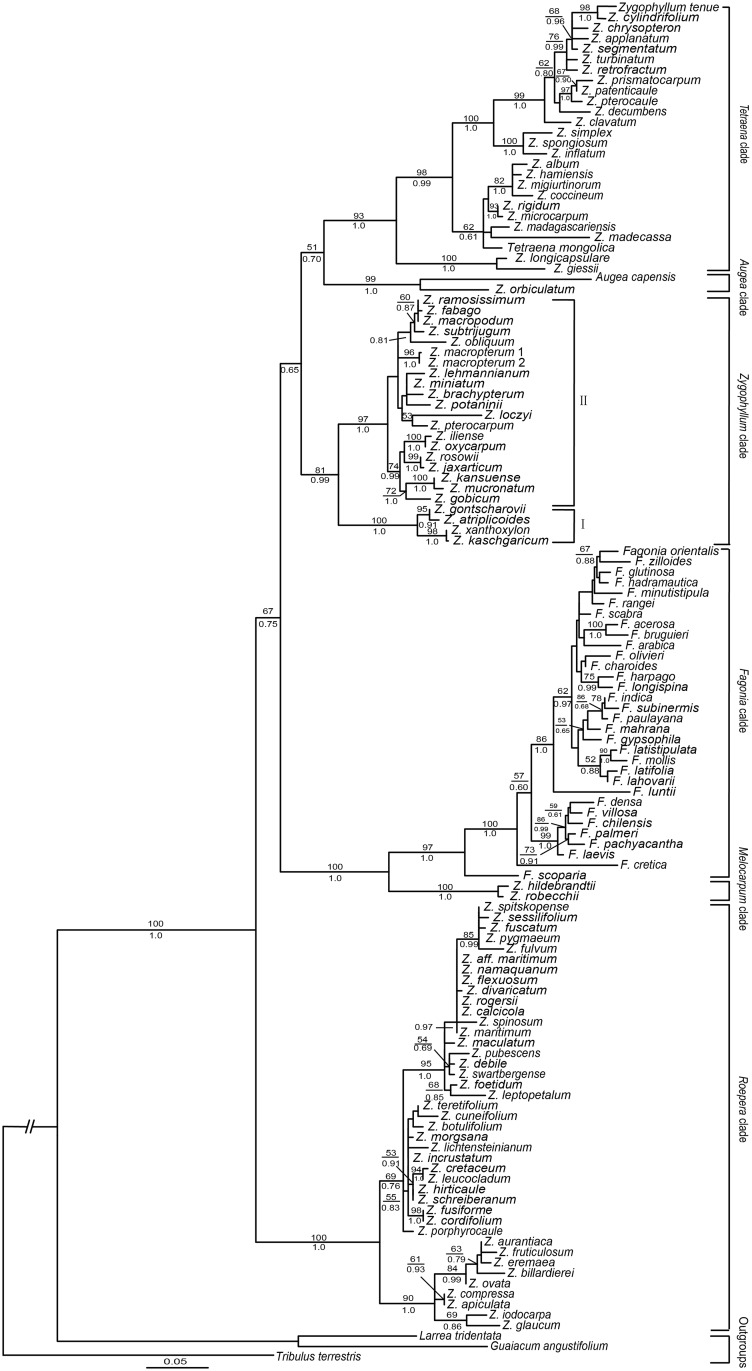
Phylogenetic relationships of Asian *Zygophyllum* and its allies obtained from an ML analysis of the three-marker dataset. Numbers above and below the branches are the bootstrap values (>50%) and Bayesian posterior probabilities (>0.50), respectively. The dash (-) indicates a node that does not appear in the BI trees. The main clades are based on Bellstedt et al., (2012).

To visualize the temporal dynamics of diversification in Asian *Zygophyllum*, a lineage-through-time (LTT) plot was constructed in the R package APE [58]. We included one of the two accessions of *Zygophyllum macropterum* in the diversification analyses, because they clustered together in the phylogenetic analysis (Fig 2). Present sampling contains only about 45% of all known Asian *Zygophyllum* species (23 of 51 species). Thus, we tested the consequence of an incomplete taxon sampling on the profile of the empirical LTT plot by generating simulated phylogenies in the R package TreeSim [59]. To ensure that the ages of the simulated trees were congruent with our real data, the speciation rates used in the simulation were calculated using the whole-clade method [60] based on the mean crown age of Asian *Zygophyllum* in the BEAST analysis (*r* = 0.166). Each simulation was generated under Yule progress with no extinction. A total of 1000 replicate phylogenies were generated containing 51 extant taxa, of which 28 were randomly pruned from each tree. The profile of LTT curves of these 1000 subsampled trees were then used to compare with the empirical LTT curve.

To examine the diversification rate change of Asian *Zygophyllum* over time, we also used the birth-death-shift model in TreePar [61], which can treat incomplete taxon sampling by setting the sampling probability (here *p*_*0*_ = 0.45). TreePar analyses were carried out with a grid setting of 0.1 million years with Yule and birth-death processes. Rate shifts were recognized as significant when *p* < 0.05 using the likelihood ratio test.

To access a quantitative method that could illustrate an overall change in diversification rate within the evolutionary history of Asian *Zygophyllum*, the rate of speciation was calculated for each lineage following the formula of Baldwin and Sanderson [62], [ln(N)–ln(N_0_)/T]. In order to assess the extinct effect on diversification rate analyses, the diversification rate under a middle relative extinction rate (ε = 0.5) and a high relative extinction rate (ε = 0.9) was also calculated based on the formula of Magallón and Sanderson [63], {log[N(1-ε) + ε]/T}.

## Results

### Phylogenetic analyses

The combined ITS, *trnL* intron and *trnL-F* dataset contains 130 taxa and 1886 characters. The tree generated by the maximum likelihood (ML) analysis was highly consistent with those retrieved from the Bayesian inference (BI) analysis (Fig 2), except for some weakly supported nodes (BS < 70%). Zygophylloideae is strongly supported as monophyletic (BS 100%, PP 1.0). Within Zygophylloideae, six major clades were identified: *Augea*, *Fagonia*, *Melocarpum*, *Tetraena*, *Zygophyllum*, and *Roepera*. The *Zygophyllum* clade contained all Asian species and was further divided into two subclades (I and II). Subclade I contained *Zygophyllum atriplicoides*, *Zygophyllum gontscharovii*, *Zygophyllum xanthoxylon*, and *Zygophyllum kaschgaricum* (BS 100%; PP 1.0), and subclade II contained all the remaining species (BS 97%, PP 1.0). Inner Mongolian *Tetraena* and some African species clustered together to form the *Tetraena* clade.

### Divergence time estimations and biogeographic analyses

**Fig 3:**
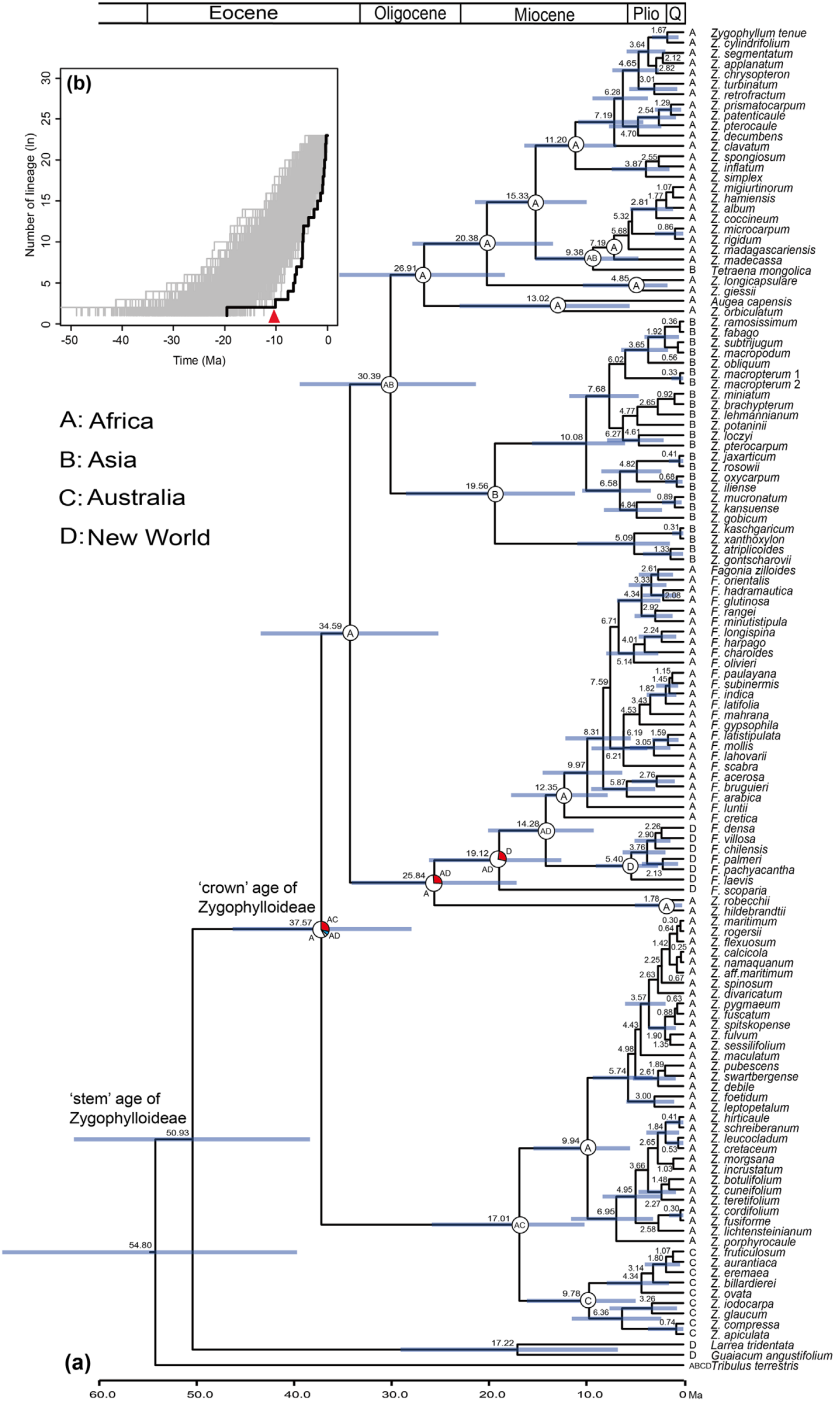
Timing, space, and tempo in the evolutionary history of Asian *Zygophyllum*. (a) Combined chronogram and biogeographic analysis of Asian *Zygophyllum*. Dating analysis was performed using BEAST software. The bars around node ages indicate the 95% highest posterior density (HPD) intervals. Large pie charts show the relative probabilities of alternative ancestral distributions obtained by Statistical Dispersal-Vicariance Analysis (S-DIVA) optimizations over the 1000 trees from the BEAST (white > red > blue > gray). (b) Lineage-through-time (LTT) plots for Asian *Zygophyllum*. The LTT plot for Asian *Zygophyllum* is indicated by a black line. The LTT plots from 1000 simulated phylogenies illustrating the effect of an incomplete sampling are shown by gray lines. The red arrow indicates the diversification rate increase located by TreePar.

The chronogram of Zygophylloideae inferred from the *rbcL* dataset is shown in S1 Fig. The stem group of Zygophylloideae occurred at 54.26 Ma (41.08–66.89 Ma, 95% highest posterior density, HPD) and the crown group diverged at 39.79 Ma (95% HPD: 29.55–51.81 Ma). Using both estimates as node priors for the combined three-marker chronogram of Zygophylloideae (Fig 3a), we obtained a similar point estimate for the crown age of Zygophylloideae (37.57 Ma, 95% HPD: 28.21–46.78 Ma). In turn, these data indicate that the stem age of Asian *Zygophyllum* was at 30.39 Ma (95% HPD: 21.53–39.81 Ma) and the crown group diverged at 19.56 Ma (95% HPD: 11.25–28.78 Ma). Inner Mongolian *Tetraena* date back to the late Miocene (9.38 Ma, 95% HPD: 5.57–15.35 Ma). These results are highly consistent with those by using the time of Magallón et al. [54] to constrain the stem age of Zygophyllaceae, in which the differences are less than 1.5 Ma. The results from using the ITS dataset also gave node ages of Zygophylloideae and Asian *Zygophyllum* that overlapped closely with those obtained from the three-marker dataset and secondary calibration points (S2 Fig).

Our biogeographic reconstruction shows that the most recent common ancestor of Zygophylloideae was likely present in Africa (Fig 3a). There were two independent dispersal events from Africa into Asia, which generated Asian *Zygophyllum* and *Tetraena*.

### Diversification rate analyses

The slope of the LTT plot obtained from the maximum clade credibility tree for Asian *Zygophyllum* is almost flat until *c*. 10 Ma and then becomes steeper (Fig 3b). Simulated LTT curves for Asian *Zygophyllum* diversity indicate a similar pattern. Interestingly, between 10 and 5 Ma, the empirical LTT plot is situated outside all the simulated 1000 LTT curves, which indicate that Asian *Zygophyllum* diversified slower than what was expected from simulations in the earlier evolutionary history, and diversified faster in the later evolutionary history

The null hypothesis of a constant diversification rate was also rejected by the TreePar analysis under Yule process in Asian *Zygophyllum* (χ^2^ = 5.4, *p* = 0.068). One significant shift in the diversification rate occurred at 10.1 Ma (Fig 3b). Initially, diversification rates of Asian *Zygophyllum* were very low (close to 0), but increased into 0.32 species per million years (myr) at 10.1 Ma. Under birth-death process, the TreePar analysis generated similar results.

**Fig 4:**
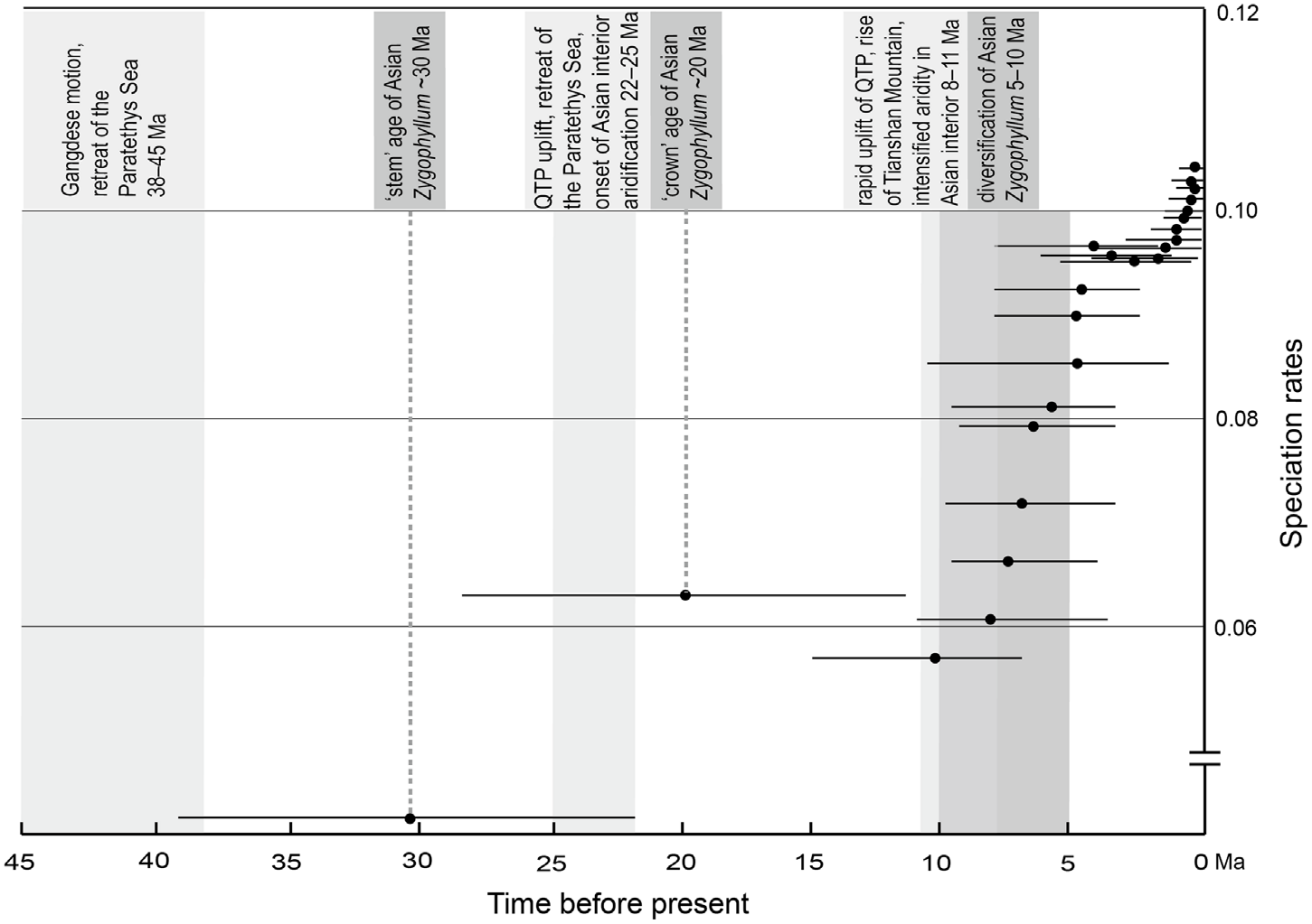
Speciation rates calculated for Asian *Zygophyllum*. Thin lines on black dots indicate the 95% highest posterior density (HPD) intervals for each node in Asian *Zygophyllum*.

Under ε = 0, the computed average speciation rate is between 0 and 0.064 species/myr between 19.56 and 10.08 Ma, and during the next 10 myr, the rates of speciation fluctuate between 0.054 and 0.105 species/myr (Fig 4). The similar results were generated under ε = 0.5 (S3 Fig) and ε = 0.9 (S4 Fig).

## Discussion

### Phylogeny

Phylogenetic analyses of the combined three-marker DNA dataset support the division of Zygophylloideae into six clades (*Augea*, *Fagonia*, *Melocarpum*, *Roepera*, *Tetraena*, and *Zygophyllum*), and that the Inner Mongolian *Tetraena* is embedded within the *Tetraena* clade. These results are in agreement with previous studies [35,42–44]. However, all these previous studies only sampled two [42–44] or nine [35] Asian *Zygophyllum* species. Our study includes data for 14 additional Asian species, and confirms that all Asian *Zygophyllum* form a monophyletic group. Based on the shrub habit and a 4-merous flower, Bunge [64] established the genus *Sarcozygium* for *S*. *xanthoxylon*. The genus was subsequently lowered to a section [65] or subgenus [66]. However, Liu [67] accepted the genus and added one species, *S*. *kaschgaricum*. At present, the majority of authors do not accept *Sarcozygium* [33,68]. Nevertheless, on the Angiosperm Phylogeny Website (http://www.mobot.org/mobot/research/apweb/), *Sarcozygium* is still listed in Zygophyllaceae [69]. Our analyses indicate that *S*. *xanthoxylon* and *S*. *kaschgaricum* and two other Asian *Zygophyllum* species, *Z*. *atriplicoides* and *Z*. *gontscharowii*, form a subclade (subclade II) with strong support (BS 100%, PP 1.0). All four species have a shrubby habit. *Zygophyllum atriplicoides* has a 5-merous flower, like the species in subclade I. Subclade I is characterized by a herbaceous habit.

### Diversification of Asian *Zygophyllum*

Our biogeographic inference indicates that Asian *Zygophyllum* and Inner Mongolian *Tetraena* originated independently from Africa, in agreement with the result of Bellstedt et al. [42]. Based on our time estimates, Asian *Zygophyllum* originated during the early Oligocene (30.39 Ma, HPD%: 21.53–39.81 Ma), which means that *Zygophyllum* successfully colonized the Asian interior from Africa at that time. The wings of *Zygophyllum* fruits are specialized for wind dispersal [33], which can facilitate long-distance dispersal. In the late Eocene (45–38 Ma), the Gangdese orogeny began to occur and resulted in the rise of the Gangdese mountains [70–72]. During the same period, the Paratethys Sea began to retreat [73], which changed the land–sea distribution. These geological events created the prerequisites for the aridification of the Asian interior [27], and could thereby have provided an opportunity for the successful colonization of the Asian interior by *Zygophyllum*.

Our time estimates indicate that Asian *Zygophyllum* became differentiated in the early Miocene (19.56 Ma, 95% HPD: 11.25–28.78 Ma). The age of the oldest eolian loess in northern China suggests that the onset of Asian interior desertification occurred at 22–25 Ma [27–29]. Studies of the eolian deposition rate and deposition fluxes indicate that the aridification of inland Asia was markedly intensified in the late Oligocene or early Miocene [27,28,74]. The uplift of the Qinghai-Tibetan Plateau (QTP) [70–72] and the continued retreat of the Paratethys Sea [26,75,76] might have been main triggers for the climatic change, which led to the expansion of arid ecosystems and consequently drove the differentiation of *Zygophyllum* in the Asian interior.

The LTT plot indicates that a dramatic increase in the number of Asian *Zygophyllum* lineages occurred during the late Miocene (*c*. 10–5 Ma; Fig 3b). Given the inferred credibility intervals of the estimated times of divergence, our calculation of speciation rates produced similar results under ε = 0 (Fig 4), 0.5 (S3 Fig), and 0.9 (S4 Fig). Our TreePar analyses detected a significant increase in the diversification rates of Asian *Zygophyllum* around 10.1 Ma (Fig 3b). These indicate that a burst of diversification of Asian *Zygophyllum* occurred during the late Miocene. Meanwhile, the Inner Mongolian *Tetraena* also originated during this period (9.38 Ma, 95% HPD: 5.57–15.35 Ma). Dettman et al. [77] and Hough et al. [78] investigated sedimentary carbonate stable isotopes in the Linxia and Xuanhua basins in the northeastern QTP and detected a shift to a more arid climate at 12–10 Ma. Zhuang et al. [79] also suggested a similar pattern for the northeastern Qaidam basin of the northern QTP. Based on the aeolian sequence containing 40 visually defined brownish-red clay and gray caliche nodule layers in the eastern Xorhol Basin, northeastern QTP, Li et al. [80] suggested that the intensified aridity of the Asian interior started between 11.5 and 10.3 Ma. Based on facies, biomarker, and stable isotopic evidence, Jian et al. [81] suggested that in the northwestern Qaidam basin, the intensified aridity occurred around 10–8 Ma.

Geological evidence suggests that there were multiple stages of uplift during the formation of the QTP [82–87], and the rapid uplift of the plateau occurred at 10–7 Ma [26,83]. From the differences in eruption time of high-K lavas in the western and eastern regions of the Qiangtang Plateau, Chung et al. [88] suggested that different locations within the QTP had different uplift histories. The significant uplift of the northern and northeastern regions of the QTP, such as Tibet, Gansu, and Qinghai, might have occurred since 15 Ma. Meanwhile, the Tianshan and Mongolia terrains also experienced considerable uplift during this period [89,90]. These orogenetic changes could have simultaneously enhanced the East Asian winter and summer monsoons and intensified the aridification of inland Asia [26]. In addition to these tectonic activities, the increase in the Eurasian land area [91] and global cooling [92,93] might also have been responsible for the middle-late Miocene aridification in Asia. The expansion of arid lands due to orogenetic and climatic changes increased the arid niche space, which might have facilitated the rapid diversification of Asian *Zygophyllum* during the late Miocene.

### Evolution of the Asian interior arid-zone biota

Geological evidence indicates that Asian inland aridity was in place in the Paleogene [23,24,94]. However, without the existence of the present high-elevation QTP, at that time arid zones occupied an extensive region in Asia, including a roughly east–west dry belt across China [23,24]. Modern Asian inland arid zones are now restricted to the interior of Eurasia, including Irano-Turania, central Asia, northwestern China, and Mongolia [21–23]. Our study shows that *Zygophyllum* successfully colonized the Asian interior from Africa in the early Oligocene (30.39 Ma, 95% HPD: 21.53–39.81 Ma), Asian *Zygophyllum* began to differentiate in the early Miocene (19.56 Ma, 95% HPD: 11.25–28.78 Ma) and experienced a burst of diversification during the late Miocene (*c*. 10 Ma). Recently, Zhang et al. [95] investigated the historical diversification of *Atraphaxis* (Polygonaceae), another dominant element of the modern Asian interior arid-zone biota, and found a similar diversification pattern. Based on the morphological pollen data from 122 sites across China, Miao et al. [96] suggested that *Artemisia* (Asteraceae), which mainly inhabits arid and semiarid regions, might have originated from the arid-semiarid middle latitudes of Asia in the late Eocene, spread west and east in the Oligocene, and became common in the western QTP during the late Miocene. Several other important elements of the modern Asian interior arid-zone biota, such as *Caragana* (Fabaceae) [97] and *Ephedra* (Ephedraceae) [98] also appear to have diversified during the middle or late Miocene. These studies indicate that the development of the Asian interior arid-zone biota was nonlinear.

Here, we propose a multi-stage evolution model, in which the Asian interior arid-zone biota originated during the late Eocene–early Oligocene, began to expand during the early Miocene, and rapidly expanded during the middle-late Miocene. *Tetraena* is restricted to arid regions of Inner Mongolia, which are located in the easternmost of Asian interior arid zone [21,23]. Our time estimates indicate that *Tetraena* originated around 10 Ma. This seems to imply that the Asian interior arid-zone biota might have expanded eastward to Inner Mongolia during the late Miocene. Yet, Asian *Zygophyllum* only consists of 51 species. This hypothesis remains to be further tested by studying more species-richness arid-adapted groups, by integrating phylogenetic, biogeographic, and molecular dating methods.

## Supporting Information

S1 Fig Chronogram of Zygophylloideae based on plastid *rbcL* sequences. The numbers in red show the locations of calibration points (see the Materials and methods section for further explanation). Bars around node ages indicate 95% highest posterior density (HPD) intervals.
(TIF)

S2 Fig Chronogram of Asian *Zygophyllum* based on nuclear ITS sequences. The numbers in red show the stem and crown ages of *Zygophllum* clade (Asian *Zygophyllum*). Bars around node ages indicate 95% highest posterior density (HPD) intervals.
(TIF)

S3 Fig Diversification rates calculated for Asian *Zygophyllum* under a middle relative extinction rate (ε = 0.5). Thin lines above black dots indicate the 95% highest posterior density (HPD) intervals for each node in Asian *Zygophyllum*.
(TIF)

S4 Fig Diversification rates calculated for Asian *Zygophyllum* under a high relative extinction rate (ε = 0.9). Thin lines above black dots indicate the 95% highest posterior density (HPD) intervals for each node in Asian *Zygophyllum*.
(TIF)

S1 Table Species and GenBank accession numbers for the data sets of three markers. (DOC)

S2 Table Species and GenBank accession numbers for the *rbcL* dataset. (DOC)

S3 Table Species and GenBank accession numbers for the ITS dataset. (DOC)
