# Supplementary figures and images for: Evolution of Asian Interior Arid-Zone Biota: Evidence from the Diversification of Asian Zygophyllum (Zygophyllaceae)
Source: PLoS One. 2015 Sep 22;10(9):e0138697. doi: 10.1371/journal.pone.0138697 (PMC4579068; doi:10.1371/journal.pone.0138697)

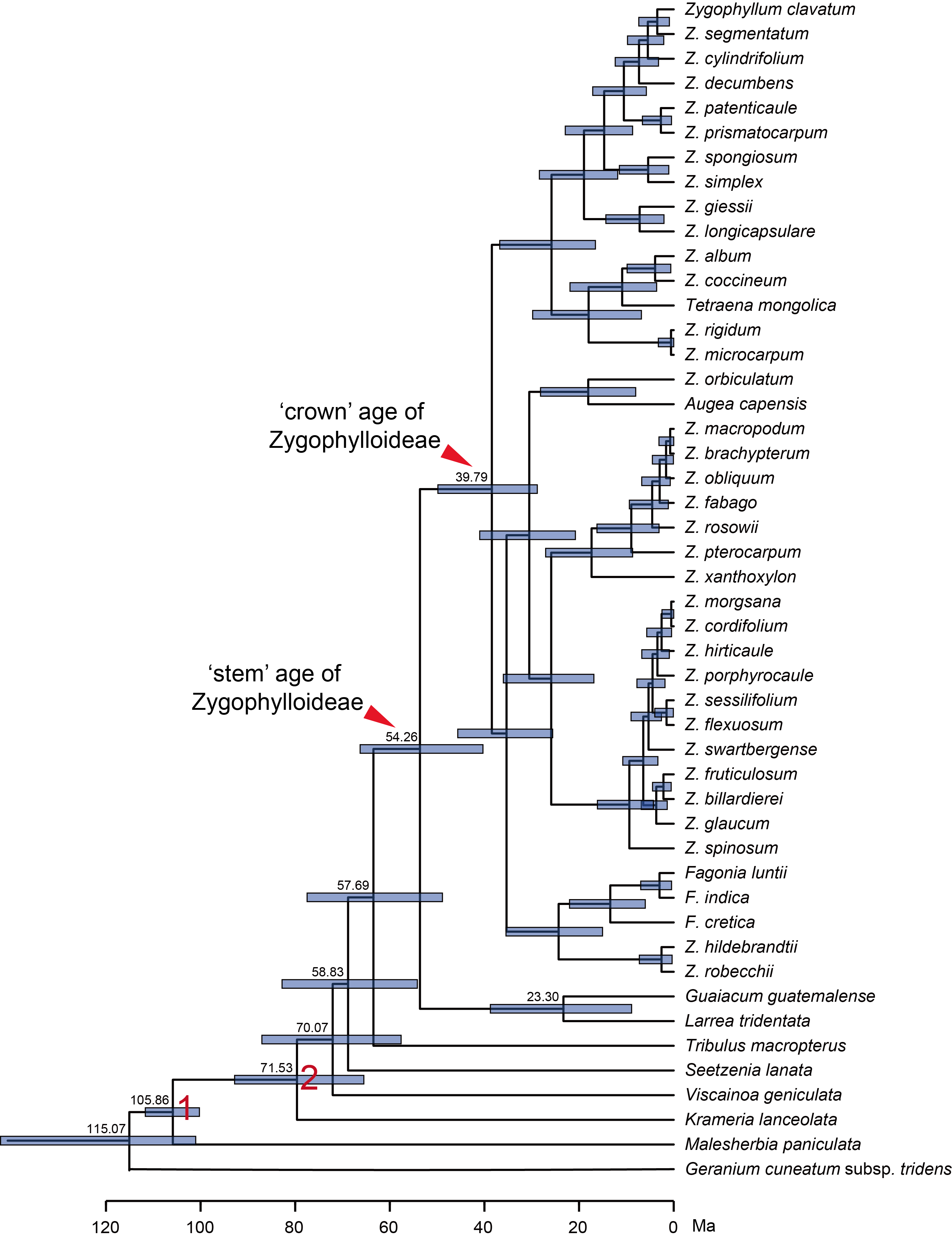

Supplement: S1 Fig — The numbers in red show the locations of calibration points (see the Materials and methods section for further explanation). Bars around node ages indicate 95% highest posterior density (HPD) intervals. (TIF) [file pone.0138697.s001.tif]

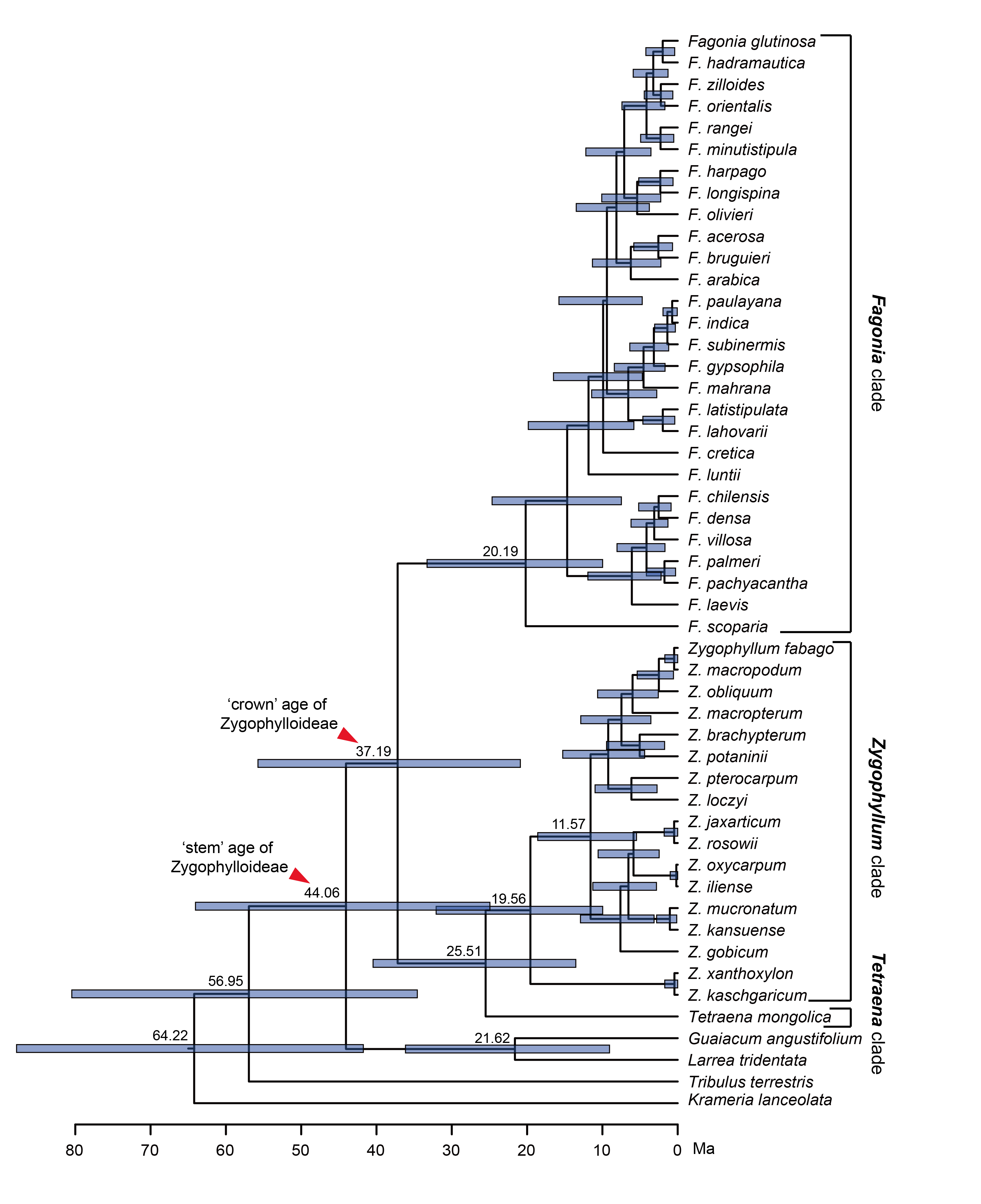

Supplement: S2 Fig — The numbers in red show the stem and crown ages of Zygophllum clade (Asian Zygophyllum). Bars around node ages indicate 95% highest posterior density (HPD) intervals. (TIF) [file pone.0138697.s002.tif]

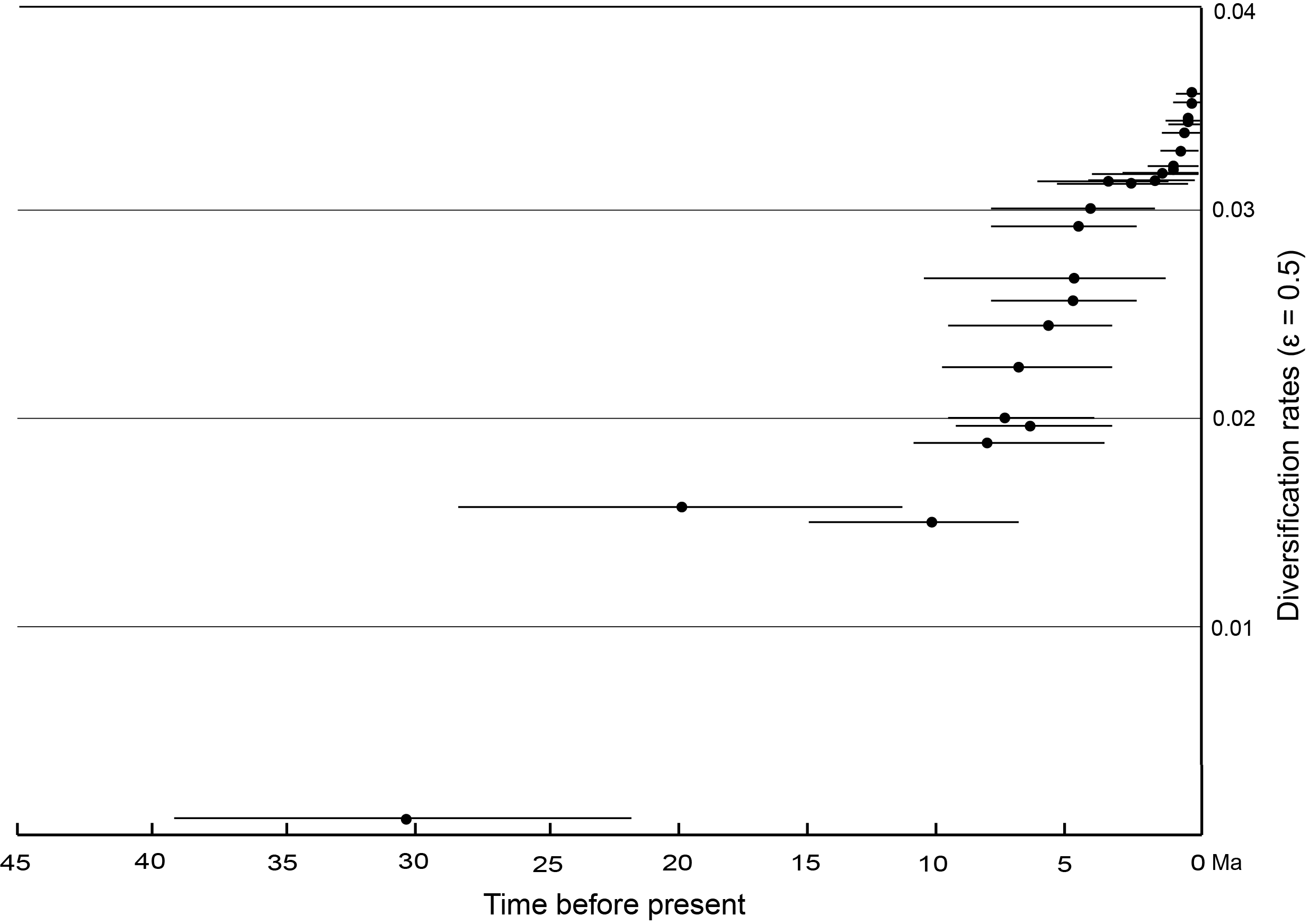

Supplement: S3 Fig — Thin lines above black dots indicate the 95% highest posterior density (HPD) intervals for each node in Asian Zygophyllum. (TIF) [file pone.0138697.s003.tif]

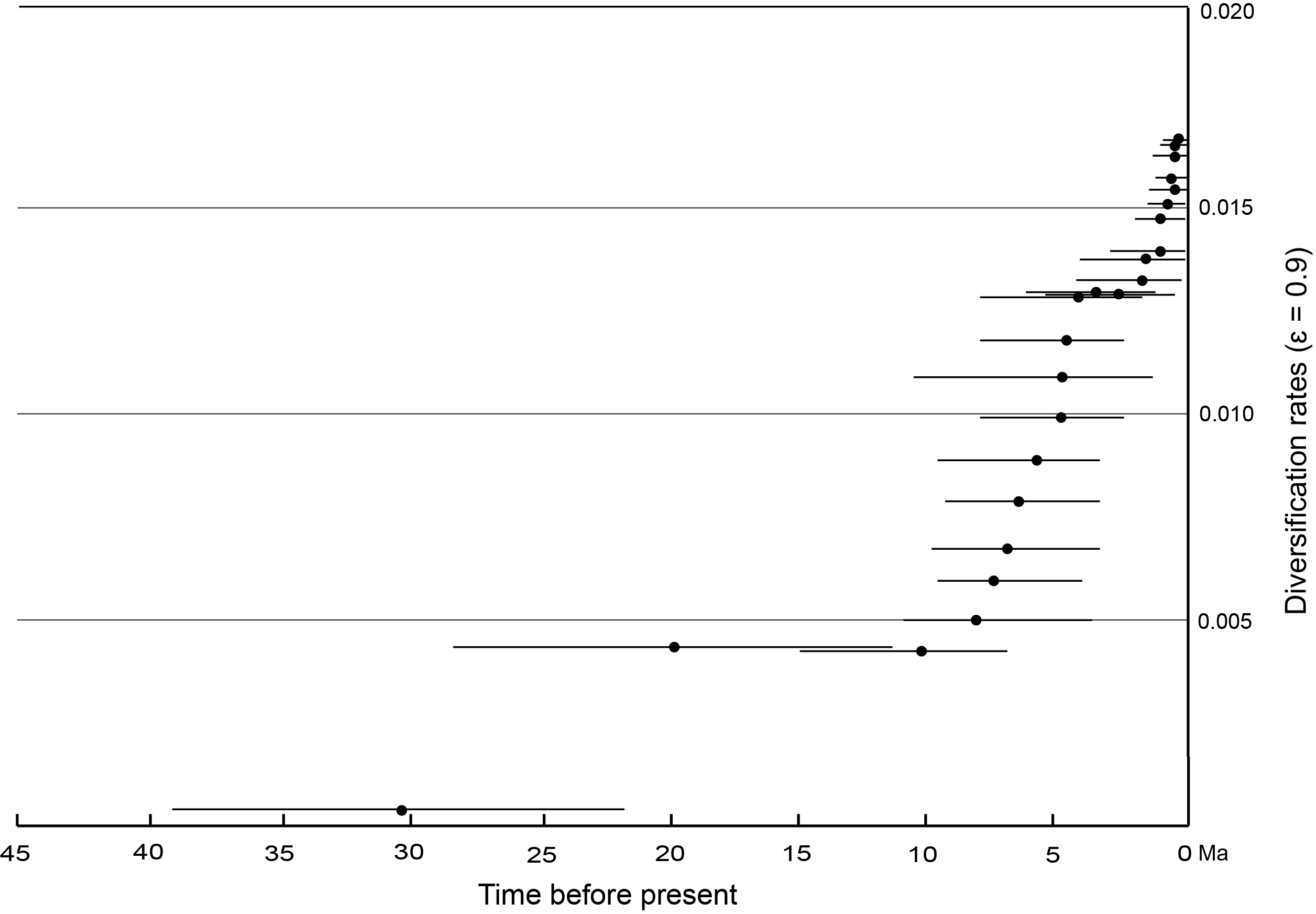

Supplement: S4 Fig — Thin lines above black dots indicate the 95% highest posterior density (HPD) intervals for each node in Asian Zygophyllum. (TIF) [file pone.0138697.s004.tif]
