# Supplementary material for: Evolution of Asian Interior Arid-Zone Biota: Evidence from the Diversification of Asian Zygophyllum (Zygophyllaceae)
Source: PLoS One. 2015 Sep 22;10(9):e0138697. doi: 10.1371/journal.pone.0138697 (PMC4579068; doi:10.1371/journal.pone.0138697)
Supplement: S1 Table — (DOC) [file pone.0138697.s005.doc]

**S1 Table. Species and GenBank accession numbers for the data sets of three markers.**

| Species | Voucher specimen | *trnL* | *trnL-F* | ITS |
| --- | --- | --- | --- | --- |
| *Zygophyllum album* L. f. | Thulin et al. 7977 (UPS) | AJ387963 | AJ387963 |  |
| *Zygophyllum aff.* *maritimum* Dold | Dold 4654 (GRA) | EF656034 | EF656034 |  |
| *Zygophyllum apiculata* F. Muell. | Greder 18664 (K) | AY233384 |  |  |
| *Zygophyllum applanatum* Van Zyl | Bellstedt 870 (STE) | EF656012 | EF656012 |  |
| *Zygophyllum atriplicoides* Fisch. & Mey. | Astanova s.n. (K) | AY233385 |  |  |
| *Zygophyllum aurantiacum* (Lindl.) F. Muell. | Greder 20900 (K) | AY300775 |  |  |
| *Zygophyllum billardierei* DC. | R. 417 (Adelaide B.G.)¹; S. R. 417 (Adelaide Botanic Garden)² | AJ387964¹ | AJ387964¹ | AY641613² |
| *Zygophyllum botulifolium* Van Zyl | Marais 451 (STE) | EF656026 | EF656026 |  |
| *Zygophyllum brachypterum* Kar. & Kir. | XJBIZLJ021 | KR001987 | KR002014 | KR002024 |
| *Zygophyllum calcicola* Van Zyl | Dreyer s.n. (STE) | EF656030 | EF656030 |  |
| *Zygophyllum chrysopteron* Retief | Marais 427 (STE) | EF656013 | EF656013 |  |
| *Zygophyllum clavatum* Schltr. & Diels | Bellstedt 878 | EF656010 | EF656010 |  |
| *Zygophyllum coccineum* L. | Ryding 1347 (K) | AJ387965 | AJ387965 |  |
| *Zygophyllum compressa* J. M. Black | Nicholls 809 (K) | AY300776 |  |  |
| *Zygophyllum cordifolium* L. f. | Marais 446 (STE) | EF656022 | EF656022 |  |
| *Zygophyllum cretaceum* Van Zyl | Bellstedt 856 (STE) | EF656028 | EF656028 |  |
| *Zygophyllum cuneifolium* Eckl. & Zeyh. | Marais 455 (STE) | EF656024 | EF656024 |  |
| *Zygophyllum cylindrifolium* Schinz | Craven 3800 (WIND) | AJ387966 | AJ387966 |  |
| *Zygophyllum debile* Cham. | Bellstedt 796 (STE) | EF656041 | EF656041 |  |
| *Zygophyllum decumbens* Delile | Thulin et al. 7981 (UPS) | AJ387967 | AJ387967 |  |
| *Zygophyllum divaricatum* Eckl. & Zeyh. | Dold 4655 (GRA) | EF656031 | EF656031 |  |
| *Zygophyllum eremaea* Diels | Beier s.n. (UPS) | AY300777 |  |  |
| *Zygophyllum fabago* L. | XJBIZLJ025 | KR001984 | KR002011 | KR002029 |
| *Zygophyllum flexuosum* Eckl. & Zeyh. | Bellstedt 794 (STE) | EF656032 | EF656032 |  |
| *Zygophyllum foetidum* Schrad. & J. C. Wendl. | Marais 423 (STE) | EF656039 | EF656039 |  |
| *Zygophyllum fruticulosum* DC. | Chase 2203 (K) | AJ387969 | AJ387969 |  |
| *Zygophyllum fulvum* L. | van Zyl 4605 (STE) | EF656044 | EF656044 |  |
| *Zygophyllum fuscatum* Van Zyl | Bellstedt 892 (STE) | EF656045 | EF656045 |  |
| *Zygophyllum fusiforme* Van Zyl | Bellstedt 857 (STE) | EF656023 | EF656023 |  |
| *Zygophyllum giessii* Merxm. & A. Schreib. | Bellstedt 874 (STE) | EF656000 | EF656000 |  |
| *Zygophyllum glaucum* F. Muell. | Chase 2204 (K) | AJ387970 | AJ387970 |  |
| *Zygophyllum gobicum* Maxim. | XJBIZLJ022 | KR001995 | KR002008 | KR002031 |
| *Zygophyllum gontscharovii* Boriss. | Astanova s.n. (K) | AY300787 |  |  |
| *Zygophyllum hamiense* Schweinf. | Thulin et al. 9840 (UPS) | AY300783 |  |  |
| *Zygophyllum hildebrandtii* Engl. | Thulin et al. 9012 (UPS) | AJ387971 | AJ387971 |  |
| *Zygophyllum hirticaule* Van Zyl | van Zyl 3894 (STE) | AJ387973 | AJ387973 |  |
| *Zygophyllum iliense* Popov | XJBIZLJ023 | KR001993 | KR002006 | KR002032 |
| *Zygophyllum incrustatum* E. Mey. ex Sond. | Bellstedt 509 (STE) | EF656019 | EF656019 |  |
| *Zygophyllum inflatum* Van Zyl | HK 1490 (WIND) | EF656005 | EF656005 |  |
| *Zygophyllum iodocarpum* F. Muell. | Symon 4607 (K) | AY300779 |  |  |
| *Zygophyllum jaxarticum* Popov | XJBIZLJ026 |  |  | KR002034 |
| *Zygophyllum kansuense* Y. X. Liou | XJBIZLJ024 | KR001996 | KR002010 | KR002022 |
| *Zygophyllum kaschgaricum* Boriss. | XJBIZLJ012 | KR001981 | KR002000 | KR002018 |
| *Zygophyllum lehmannianum* Bunge | June s.n. 1972 (K) | AY300788 |  |  |
| *Zygophyllum leptopetalum* E. Mey. ex Sond. | Marais 422 (STE) | EF656040 | EF656040 |  |
| *Zygophyllum leucocladum* Diels | van Zyl 4479 (STE) | EF656029 | EF656029 |  |
| *Zygophyllum lichtensteinianum* Cham. | Van Zyl 4594 (STE) | EF656020 | EF656020 |  |
| *Zygophyllum loczyi* Kanitz | XJBIZLJ019 | KR001988 | KR002015 | KR002021 |
| *Zygophyllum longicapsulare* Schinz | Bellstedt 879 (STE) | EF656001 | EF656001 |  |
| *Zygophyllum macropodum* Boriss. | XJBIZLJ001 |  |  | KR002030 |
| *Zygophyllum macropterum*^1^ C. A. Mey. | XJBIZLJ017 | KR001991 | KR002003 | KR002026 |
| *Zygophyllum macropterum*^2^ var. microphyllum Boriss. | XJBIZLJ018 | KR001990 | KR002004 | KR002027 |
| *Zygophyllum maculatum* Aiton | Marais 433 (STE) | EF656033 | EF656033 |  |
| *Zygophyllum madagascariensis* (Baill.) Stauffer | Keating Miller 2236 (K) | AY300784 |  |  |
| *Zygophyllum madecassum* H. Perrier | Lorence s.n. (K) | AY300785 |  |  |
| *Zygophyllum maritimum* Eckl. & Zeyh. | Dold 4656 (GRA) | EF656035 | EF656035 |  |
| *Zygophyllum microcarpum* E. Mey. | van Zyl 4591 (STE) | EF656002 | EF656002 |  |
| *Zygophyllum migiurtinorum* Chiov. | Thulin et al. 9553 (UPS) | AY300786 |  |  |
| *Zygophyllum miniatum* Cham. | June s.n. 1965 (K) | AY300789 |  |  |
| *Zygophyllum morgsana* L. | Bellstedt 890 (STE) | EF656021 | EF656021 |  |
| *Zygophyllum mucronatum* Maxim. | XJBIZLJ030 | KR001997 | KR002009 | KR002023 |
| *Zygophyllum namaquanum* Van Zyl | Marais 440 (STE) | EF656036 | EF656036 |  |
| *Zygophyllum obliquum* Popov | XJBIZLJ028 | KR001989 | KR002002 | KR002028 |
| *Zygophyllum orbiculatum* Welw. ex Oliv. | Craven 5096 (WIND) | EF655999 | EF655999 |  |
| *Zygophyllum ovatum* Ewart & J. White | Melville 451 (K) | AY300782 |  |  |
| *Zygophyllum oxycarpum* Popov | XJBIZLJ031 | KR001992 | KR002007 | KR002033 |
| *Zygophyllum patenticaule* Van Zyl | Bellstedt 868 (STE) | EF656008 | EF656008 |  |
| *Zygophyllum porphyrocaule* Van Zyl | Bellstedt 800 (STE) | EF656018 | EF656018 |  |
| *Zygophyllum potaninii* Maxim. | XJBIZLJ020 | KR001986 | KR002013 | KR002020 |
| *Zygophyllum prismatocarpum* Sond. | Bellstedt 860 (STE) | EF656009 | EF656009 |  |
| *Zygophyllum pterocarpum* Bunge | XJBIZLJ016 | KR001985 | KR002012 | KR002025 |
| *Zygophyllum pterocaule* Van Zyl | Mucina 270806/25 (STE) | EF656007 | EF656007 |  |
| *Zygophyllum pubescens* Schinz | Bellstedt 881 (STE) | EF656042 | EF656042 |  |
| *Zygophyllum pygmaeum* Eckl. & Zeyh. | Marais 424 (STE) | EF656046 | EF656046 |  |
| *Zygophyllum ramosissimum* Popov | Granitov s.n. (K) | AY300790 |  |  |
| *Zygophyllum retrofractum* Thunb. | Marais 430 (STE) | EF656014 | EF656014 |  |
| *Zygophyllum rigidum* Schinz | van Zyl 4590 (STE) | EF656003 | EF656003 |  |
| *Zygophyllum robecchii* Engl. | Chase 636 (K) | AJ387972 | AJ387972 |  |
| *Zygophyllum rogersii* Compton | Marais 432 (STE) | EF656037 | EF656037 |  |
| *Zygophyllum rosowii* Bunge | XJBIZLJ027 | KR001994 | KR002005 | KR002035 |
| *Zygophyllum schreiberanum* Merxm. & Giess | Bellstedt 871 (STE) | EF656027 | EF656027 |  |
| *Zygophyllum segmentatum* Van Zyl | Bellstedt 861 (STE) | EF656015 | EF656015 |  |
| *Zygophyllum sessilifolium* L. | Marais 434 (STE) | EF656047 | EF656047 |  |
| *Zygophyllum simplex* L. | Chase 806 (K) | AJ387974 | AJ387974 |  |
| *Zygophyllum spinosum* L. | Bellstedt 801 (STE) | EF656038 | EF656038 |  |
| *Zygophyllum spitskopense* Van Zyl | van Zyl 4606 (STE) | EF656048 | EF656048 |  |
| *Zygophyllum spongiosum* Van Zyl | HK 1573 (WIND) | EF656006 | EF656006 |  |
| *Zygophyllum subtrijugum* C. A. Mey. | 1955.07.30 s. leg. s.n. (K) | AY300792 |  |  |
| *Zygophyllum swartbergense* Van Zyl | Bellstedt 798 (STE) | EF656043 | EF656043 |  |
| *Zygophyllum tenue* R. Glover | van Zyl 4593 (STE) | EF656017 | EF656017 |  |
| *Zygophyllum teretifolium* Schltr. | Marais 447 (STE) | EF656025 | EF656025 |  |
| *Zygophyllum turbinatum* Van Zyl | Bellstedt 799 (STE) | EF656016 | EF656016 |  |
| *Zygophyllum xanthoxylum* (Bunge) Maxim. | XJBIZLJ013 | KR001982 | KR002001 | KR002019 |
| *Fagonia acerosa* Boiss. | Davis 56261 (E) | AY641579 |  | AY641617 |
| *Fagonia arabica* L. | Leonard 4887 (S) | AY641580 |  | AY641618 |
| *Fagonia bruguieri* DC. | Thulin et al. 9986 (UPS) | AY641582 |  | AY641619 |
| *Fagonia charoides* Chiov. | Thulin et al. 10587 (UPS) | AY641583 |  | AY641621 |
| *Fagonia chilensis* Hook. & Arn. | Penailillo s.n. (UTALCA) | AY641584 |  | AY641622 |
| *Fagonia cretica* L. | Chase 3432 K¹; Beier 125 (UPS)² | AJ387942¹ | AJ387942¹ | AY641623² |
| *Fagonia densa* I. M. Johnst. | Rebman 3171 (SD) | AY641587 |  | AY641625 |
| *Fagonia glutinosa* Delile | Davis 49654 (K) | AY641588 |  | AY641627 |
| *Fagonia gypsophila* Beier & Thulin | Thulin et al. 9473 (UPS) | AY641589 |  | AY641626 |
| *Fagonia hadramautica* Beier & Thulin | Thulin et al. 9808 (UPS) | AY641590 |  | AY641628 |
| *Fagonia harpago* Emb. & Maire | Podlech 40630 (RSA) | AY641591 |  | AY641629 |
| *Fagonia indica* Burm. f. | Thulin et al. 9835 (UPS) | AY300769 | AJ387943 | AY641630 |
| *Fagonia laevis* Standl. | Beier 97 (UPS) | AY641595 |  | AY641633 |
| *Fagonia lahovarii* Volkens & Schweinf. | Thulin et al. 9522 (UPS) | AY641596 |  | AY641635 |
| *Fagonia latifolia* Delile | Scholz 174 (B) | AY641597 |  |  |
| *Fagonia latistipulata* Beier & Thulin | Thulin et al. 10833 (UPS) | AY641598 |  | AY641636 |
| *Fagonia longispina* Batt. | Podlech 53369 (M) | AY641599 |  | AY641637 |
| *Fagonia luntii* Baker | Thulin et al. 9881 (UPS) | AJ387944 | AJ387944 | AY641638 |
| *Fagonia mahrana* Beier | Thulin et al. 9682 (UPS) | AY641600 |  | AY641639 |
| *Fagonia minutistipula* Engl. | Giess and Müller 13952 (K) | AY300771 |  | AY641641 |
| *Fagonia mollis* Delile | Townsend 86/12 (K) | AY641601 |  | AY641643 |
| *Fagonia olivieri* DC. | Samuelsson 4357 (S) | AY641602 |  | AY641646 |
| *Fagonia orientalis* C. Presl | Collenette 7516 (E) | AY641603 |  | AY641648 |
| *Fagonia pachyacantha* Rydb. | Beier 93 (UPS) | AY641604 |  | AY641649 |
| *Fagonia palmeri* Vasey & Rose | Hastings 75 (SD) | AY641605 |  | AY641653 |
| *Fagonia paulayana* J. Wagner & Vierh. | Thulin et al. 9515 (UPS) | AY641608 |  | AY641654 |
| *Fagonia rangei* Loes. ex Engl. | Leistner 3388 (K) | AY641609 |  | AY641647 |
| *Fagonia scabra* Forssk. | Davis 49662 (E) | AY300768 |  | AY641645 |
| *Fagonia scoparia* Brandegee | Johnston 9461 (SD) | AY300773 |  | AY641644 |
| *Fagonia subinermis* Boiss. | Grey-Wilson and Hewer 285 (W) | AY641610 |  | AY641642 |
| *Fagonia villosa* D. M. Porter | K. 5915 (RSA) | AY641611 |  | AY641640 |
| *Fagonia zilloides* Humbert | Davis 49047 (E) | AY641612 |  | AY641655 |
| *Augea capensis* Thunb. | Bellstedt 934 (STE) | EF655998 |  |  |
| *Tetraena mongolica* Maxim. | XJBIZLJ015 | KR001983 | KR001999 | KR002017 |
| *Guaiacum angustifolium* Engelm. | DEK: J. R. Dertien 534 (unpublished) | EU253465 | EU253465 | JX486127 |
| *Larrea tridentata* (Sessé & Moc. ex DC.) Coville | Chase 636 (K)¹;  R. Laport 766242 (RSA)² | AJ387951¹ | AJ387951¹ | JF267306² |
| *Tribulus terrestris* L. | XJBIZLJ014 | KR001980 | KR001998 | KR002016 |
