# Supplementary material for: Evolution of Asian Interior Arid-Zone Biota: Evidence from the Diversification of Asian Zygophyllum (Zygophyllaceae)
Source: PLoS One. 2015 Sep 22;10(9):e0138697. doi: 10.1371/journal.pone.0138697 (PMC4579068; doi:10.1371/journal.pone.0138697)
Supplement: S2 Table — (DOC) [file pone.0138697.s006.doc]

**S2 Table. Species and GenBank accession numbers for the *rbcL* dataset.**

| Species | Voucher | *rbcL* |
| --- | --- | --- |
| *Zygophyllum album* L. | Thulin et al. 7977 (UPS) | AJ133861 |
| *Zygophyllum billardierei* DC. | S. R. 417 (Adelaide Botanic Garden) | AJ133862 |
| *Zygophyllum brachypterum* Kar. & Kir. | Li ZJ 0850 | JF944800 |
| *Zygophyllum clavatum* Schltr. & Diels | Bellstedt 878 (STE) | EF655986 |
| *Zygophyllum coccineum* L. | Ryding 1347 (K) | AJ133863 |
| *Zygophyllum cordifolium* L. f. | Marais 446 (STE) | EF655993 |
| *Zygophyllum cylindrifolium* Schinz | Craven 3800 (WIND) | AJ133864 |
| *Zygophyllum decumbens* Delile | Thulin et al. 7981 (UPS) | AJ133865 |
| *Zygophyllum fabago* L. | Chase 516 (K) | Y15030 |
| *Zygophyllum flexuosum* Eckl. & Zeyh. | Bellstedt 794 (STE) | EF655995 |
| *Zygophyllum fruticulosum* DC. | Chase 2203 (K) | AJ133866 |
| *Zygophyllum giessii* Merxm. & A. Schreib. | Bellstedt 874 (STE) | EF655980 |
| *Zygophyllum glaucum* E. Mey. ex Sond. | Chase 2204 (K) | AJ133867 |
| *Zygophyllum hildebrandtii* Engl. | Thulin et al. 9012 (UPS) | AJ133868 |
| *Zygophyllum hirticaule* Van Zyl | Van Zyl 3894 (STE) | AJ133869 |
| *Zygophyllum longicapsulare* Schinz | Bellstedt 879 (STE) | EF655981 |
| *Zygophyllum macropodum* Boriss. | Liu JQ 0049 | JF944806 |
| *Zygophyllum microcarpum* Licht. ex Cham. | van Zyl 4591 (STE) | EF655983 |
| *Zygophyllum morgsana* L. | Bellstedt 890 (STE) | EF655994 |
| *Zygophyllum obliquum* Popov | Li ZJ 0354 | JF944808 |
| *Zygophyllum orbiculatum* Welw. ex Oliver | Craven 5096 (WIND) | EF655979 |
| *Zygophyllum patenticaule* Van Zyl | Bellstedt 868 (STE) | EF655989 |
| *Zygophyllum porphyrocaule* Van Zyl | Bellstedt 800 (STE) | EF655992 |
| *Zygophyllum prismatocarpum* Sond. | Bellstedt 860 (STE) | EF655990 |
| *Zygophyllum pterocarpum* Bunge | Tan DY 0003 | JF944809 |
| *Zygophyllum rigidum* Schinz | van Zyl 4590 (STE) | EF655982 |
| *Zygophyllum robecchii* Engl. | Thulin et al. 8428 (UPS) | AJ133870 |
| *Zygophyllum rosowii* Bunge | Zhang DY 153 | JF944811 |
| *Zygophyllum segmentatum* Van Zyl | Bellstedt 861 (STE) | EF655987 |
| *Zygophyllum sessilifolium* L. | Marais 434 (STE) | EF655997 |
| *Zygophyllum simplex* L. | Chase 806 (K) | Y15031 |
| *Zygophyllum spinosum* L. | Viviers 426 (K) | AJ133871 |
| *Zygophyllum spongiosum* Van Zyl | HK 1573 (WIND) | EF655985 |
| *Zygophyllum swartbergense* Van Zyl | Bellstedt 798 (STE) | EF655996 |
| *Zygophyllum xanthoxylum* (Bunge) Maxim. | Chase 1700 (K) | AJ133872 |
| *Fagonia cretica* L. | Chase 3432 (K) | AJ133855 |
| *Fagonia luntii* Bak. | Wieland 4504 (K) | AJ133856 |
| *Fagonia indica* Burm. f. | Collenette 10/93 (K) | Y15018 |
| *Augea capensis* Thunb. | Bellstedt 934 (STE) | EF655978 |
| *Larrea tridentata* (Sessé & Moc. ex DC.) Coville | Chase 636 (K) | Y15022 |
| *Tetraena mongolica* Maxim. | Shenhan 1994 (K) | Y15027 |
| *Guaiacum guatemalense* Planch. ex Rydb. & Vail | Chase 640 (K) | Y15019 |
| *Tribulus macropterus* Boiss. | Wilson 4719 (NSW) | Y15028 |
| *Seetzenia lanata* (Willd.) Bullock | Herman 3964 (K) | Y15025 |
| *Krameria lanceolata* Torr. | Simpson 88-05-1-1 (MICH) | Y15032 |
| *Viscainoa geniculata* (Kellogg) Greene | Chase 634 (K) | Y15029 |
| *Malesherbia paniculata* D. Don | Zollner 5291 (2009, unpublished) | AB536558 |
| *Geranium cuneatum* subsp. *tridens* (Hillebr.) Carlquist & Bissing | Pax & Michaels HH92-9 | U77903 |
