# Supplementary material for: Evolution of Asian Interior Arid-Zone Biota: Evidence from the Diversification of Asian Zygophyllum (Zygophyllaceae)
Source: PLoS One. 2015 Sep 22;10(9):e0138697. doi: 10.1371/journal.pone.0138697 (PMC4579068; doi:10.1371/journal.pone.0138697)
Supplement: S3 Table — (DOC) [file pone.0138697.s007.doc]

**S3 Table. Species and GenBank accession numbers for the ITS dataset.**

| Species | Voucher | *rbcL* |
| --- | --- | --- |
| *Zygophyllum billardierei* DC. | S. R. 417 (Adelaide Botanic Garden) | AY641613 |
| *Zygophyllum brachypterum* Kar. & Kir. | XJBIZLJ021 | KR002024 |
| *Zygophyllum fabago* L. | XJBIZLJ025 | KR002029 |
| *Zygophyllum gobicum* Maxim. | XJBIZLJ022 | KR002031 |
| *Zygophyllum iliense* Popov | XJBIZLJ023 | KR002032 |
| *Zygophyllum jaxarticum* Popov | XJBIZLJ026 | KR002034 |
| *Zygophyllum kansuense* Y. X. Liou | XJBIZLJ024 | KR002022 |
| *Zygophyllum kaschgaricum* Boriss. | XJBIZLJ012 | KR002018 |
| *Zygophyllum loczyi* Kanitz | XJBIZLJ019 | Y15030 |
| *Zygophyllum macropodum* Boriss. | XJBIZLJ001 | KR002030 |
| *Zygophyllum macropterum*^1^ C. A. Mey. | XJBIZLJ017 | KR002026 |
| *Zygophyllum mucronatum* Maxim. | XJBIZLJ030 | KR002023 |
| *Zygophyllum obliquum* Popov | XJBIZLJ028 | KR002028 |
| *Zygophyllum oxycarpum* Popov | XJBIZLJ031 | KR002033 |
| *Zygophyllum potaninii* Maxim. | XJBIZLJ020 | KR002020 |
| *Zygophyllum pterocarpum* Bunge | XJBIZLJ016 | KR002025 |
| *Zygophyllum rosowii* Bunge | XJBIZLJ027 | KR002035 |
| *Zygophyllum xanthoxylum* (Bunge) Maxim. | XJBIZLJ013 | KR002019 |
| *Fagonia acerosa* Boiss. | Bellstedt 890 (STE) | AY641617 |
| *Fagonia arabica* L. | Li ZJ 0354 | AY641618 |
| *Fagonia bruguieri* DC. | Craven 5096 (WIND) | AY641619 |
| *Fagonia charoides* Chiov. | Bellstedt 868 (STE) | AY641621 |
| *Fagonia chilensis* Hook. & Arn. | Bellstedt 800 (STE) | AY641622 |
| *Fagonia cretica* L. | Bellstedt 860 (STE) | AY641623² |
| *Fagonia densa* I. M. Johnst. | Tan DY 0003 | AY641625 |
| *Fagonia glutinosa* Delile | van Zyl 4590 (STE) | AY641627 |
| *Fagonia gypsophila* Beier & Thulin | Thulin et al. 8428 (UPS) | AY641626 |
| *Fagonia hadramautica* Beier & Thulin | Zhang DY 153 | AY641628 |
| *Fagonia harpago* Emb. & Maire | Bellstedt 861 (STE) | AY641629 |
| *Fagonia indica* Burm. f. | Marais 434 (STE) | AY641630 |
| *Fagonia laevis* Standl. | Chase 806 (K) | AY641633 |
| *Fagonia lahovarii* Volkens & Schweinf. | Viviers 426 (K) | AY641635 |
| *Fagonia latistipulata* Beier & Thulin | HK 1573 (WIND) | AY641636 |
| *Fagonia longispina* Batt. | Bellstedt 798 (STE) | AY641637 |
| *Fagonia luntii* Baker | Chase 1700 (K) | AY641638 |
| *Fagonia mahrana* Beier | Chase 3432 (K) | AY641639 |
| *Fagonia minutistipula* Engl. | Wieland 4504 (K) | AY641641 |
| *Fagonia mollis* Delile | Collenette 10/93 (K) | AY641643 |
| *Fagonia olivieri* DC. | Bellstedt 934 (STE) | AY641646 |
| *Fagonia orientalis* C. Presl | Chase 636 (K) | AY641648 |
| *Fagonia pachyacantha* Rydb. | Shenhan 1994 (K) | AY641649 |
| *Fagonia palmeri* Vasey & Rose | Chase 640 (K) | AY641653 |
| *Fagonia paulayana* J. Wagner & Vierh. | Wilson 4719 (NSW) | AY641654 |
| *Fagonia rangei* Loes. ex Engl. | Herman 3964 (K) | AY641647 |
| *Fagonia scabra* Forssk. | Simpson 88-05-1-1 (MICH) | AY641645 |
| *Fagonia scoparia* Brandegee | Chase 634 (K) | AY641644 |
| *Fagonia subinermis* Boiss. | Zollner 5291 (2009, unpublished) | AY641642 |
| *Fagonia villosa* D. M. Porter | Pax & Michaels HH92-9 | AY641640 |
| *Fagonia zilloides* Humbert | Davis 49047 (E) | AY641655 |
| *Tetraena mongolica* Maxim. | XJBIZLJ015 | KR002017 |
| *Guaiacum angustifolium* Engelm. | DEK: J. R. Dertien 534 (unpublished) | JX486127 |
| *Larrea tridentata* (Sessé & Moc. ex DC.) Coville | R. Laport 766242 (RSA) | JF267306 |
| *Tribulus terrestris* L. | XJBIZLJ014 | KR002016 |
| *Krameria lanceolata* Torr. | E. L. Bridges & K. Kindscher 13622 | AY261080 |
